# Efficiency and Applicability of Virtual Surgical Planning in Maxillofacial and Mandibular Bone Reconstruction: A Narrative Review

**DOI:** 10.3390/clinpract15030062

**Published:** 2025-03-14

**Authors:** Mohammed Mahmoud Shalabi, Khaldoun M. A. Darwich, Mohammad Naem Kheshfeh, Mohammad Younis Hajeer

**Affiliations:** 1 Department of Oral and Maxillofacial Surgery, Faculty of Dentistry, University of Damascus, Damascus P.O. Box 30621, Syria; mohammed86.shalabi@damascusuniversity.edu.sy (M.M.S.); khaldoun.darwich@damascusuniversity.edu.sy (K.M.A.D.); 2 Department of Orthodontics, Faculty of Dentistry, University of Damascus, Damascus P.O. Box 30621, Syria; mohammad.kheshfeh@damascusuniversity.edu.sy

**Keywords:** computer-assisted surgery, mandibular reconstruction, maxillofacial surgery, review, stereolithography, surgical simulation

## Abstract

**Background:** Facial structures are critical to aesthetics and function. Deformities can cause significant problems. Advances in surgical techniques, including three-dimensional (3D) computer simulation and virtual surgical planning (VSP), have improved outcomes. VSP accurately predicts surgical outcomes, revolutionizing facial reconstruction. This article reviews VSP in facial bone reconstruction, highlighting its advantages and accuracy over traditional methods. **Methods:** A systematic search using Medline (PubMed), Web of Science, Scopus, and Google Scholar revealed 1645 articles that addressed the topic of this study. **Results:** The systematic search yielded 64 articles that were highly relevant to the study objective, underscoring the critical importance of virtual surgical planning (VSP) in enhancing surgical precision and patient satisfaction. VSP has become a key player in improving surgical interventions and reducing complications, reinforcing its role as the preferred method in modern reconstructive surgery and thus improving functional and aesthetic outcomes, significantly enhancing patient satisfaction, and ensuring accurate interpretation of treatment plans. When compared to traditional surgical planning (TSP), VSP offers increased accuracy, shorter operating times, and superior aesthetic outcomes. **Conclusions:** VSP has been shown to effectively manage the complex challenges of facial anatomy and has significantly enhanced the planning and execution of reconstructive surgeries. This has been achieved by leveraging advanced imaging and computer-aided design.

## 1. Introduction

Facial deformities resulting from trauma or cancer resections pose significant physiological and psychological challenges. Beyond being stigmatizing and aesthetically displeasing, inadequate repair of facial deformities can severely impact essential functions such as respiration, mastication, speaking, and swallowing [1]. Facial deformities not only affect physical appearance but also have profound effects on psychological well-being, often leading to social isolation and decreased quality of life [2]. Facial bone reconstruction surgery aims to restore the appearance and function of the facial bones, often necessary for patients with severe injuries, cancer, or congenital deformities. The primary goal is to repair damaged tissues, thereby improving both the aesthetic and functional aspects of the face [3,4]. These surgeries address facial fractures, bone defects, clefts, skeletal malocclusions, and cancer-affected bone tissue. Consequently, reconstructive techniques continually evolve to support treatment while maintaining postoperative quality of life. Microvascular free tissue transfer became widely popular in the 1970s due to the introduction of smaller sutures, which were essential for precise re-anastomosis [5]. Facial reconstructive procedures are diverse and tailored to address various conditions, including trauma, congenital deformities, and post-cancer resections [6]. These procedures include facial trauma surgery, which repairs fractures and other injuries using plates, screws, or wires to stabilize bones; cleft lip and palate repair, which corrects congenital conditions to improve appearance and function; rhinoplasty for reshaping the nose; ear reconstruction to reshape the outer ear; scar removal through laser treatments or surgical excision; facial reconstruction after cancer surgery using grafts or flaps; facial reanimation to restore movement in paralyzed facial muscles; chin augmentation to reshape the chin; skin grafts to cover defects; microvascular free tissue transfer to transfer skin, bone, or muscle with tiny blood vessels reconnected; local tissue rearrangement to use skin flaps from one part of the face to cover another part; and implants or prosthetics made from materials like ceramics or plastic for facial features [6]. Subsequent advancements involved the effective use of osteocutaneous free tissue transfer, incorporating donor bones from the fibula, iliac crest, and scapular flap to address maxillary and mandibular defects [7]. Grafts can include skin grafts (full-thickness or split-thickness), composite grafts (multiple tissue types), bone grafts (from another part of the body or donor), and soft tissue grafts (fat, muscle, or other soft tissues) [6]. Advantages of these procedures include improved functionality, enhanced appearance, reduced pain and discomfort, and natural results. However, disadvantages can include the risk of rejection, infection, scarring, high cost, and long recovery times [8]. Despite these challenges, facial reconstructive procedures continue to evolve, incorporating advanced imaging technologies and virtual surgical planning (VSP) to enhance precision and outcomes [6,9].

Nevertheless, these surgeries are technically demanding and necessitate meticulous planning to restore form and function [3]. Inadequate pre-operative planning can greatly jeopardize these outcomes. Therefore, a strong focus on enhancing pre-operative planning has led surgeons to develop innovative reconstructive techniques. Traditionally, reconstructions were performed using free-hand surgery (FHS), where one or more donor bone segments were shaped to fit the defect. Recently, VSP has become widely available through commercial or open-source software, improving outcomes. The introduction of VSP has revolutionized the field by allowing surgeons to simulate and refine surgical procedures before performing them on patients [10]. VSP encompasses a variety of processes, including planning tumor resection margins, optimizing donor site harvests, creating patient-specific resection or harvest cutting guides, and pre-bending or manufacturing patient-specific reconstruction plates [11]. Advancements in imaging technologies, such as CT and MRI scans, have greatly enhanced the precision of pre-operative planning, allowing for more accurate assessments of facial structures [12]. The execution of facial epitheses in reconstructive surgery involves several steps. First, a thorough assessment of the patient’s condition is conducted, including medical history, physical examination, and imaging studies. A custom-made epithesis is then created from medical-grade silicone and tailored to the patient’s skin. Initially, an adhesive epithesis serves as an interim solution while the patient undergoes necessary adjuvant therapy. Once the wound has healed, platinum implants with magnetic pins are inserted into the bone at the defect’s edge under general anaesthesia. After healing, the final epithesis is fabricated and placed on the magnetic pins, allowing for easy removal for cleaning and maintenance. This method provides a non-invasive, aesthetically pleasing solution, improving both functionality and appearance [13,14].

In facial reconstructive surgery, individual implants can be categorized into three main types: milled, sintered, and printed implants [15]. Milled implants are custom-made using computer-aided design (CAD) and computer-aided manufacturing (CAM) technologies, where a block of material, often titanium or PEEK (polyether ether ketone), is milled to create the precise shape and size required for the patient’s specific needs. Sintered implants involve the use of powdered materials that are heated and compressed to form a solid implant, allowing for the creation of complex shapes and structures that are difficult to achieve with traditional manufacturing techniques. Additive manufacturing, or three-dimensional (3D) printing, is a rapidly growing technology in facial reconstructive surgery [15]. This technique involves layering materials, such as titanium or biocompatible polymers, to create implants that are highly customized to the patient’s anatomy. Additionally, 3D printing allows for intricate designs and rapid production of implants. Each type of implant has its advantages and disadvantages: milled implants provide high precision and strength but can be more expensive and time-consuming to produce; sintered implants offer flexibility in design and material choice but may have lower mechanical strength compared to milled implants; and 3D-printed implants allow for rapid customization and complex geometries, but the technology and materials used may still be evolving [15].

As facial bone reconstruction has evolved, so have photographic techniques for patient evaluation, surgical planning, and follow-up [8]. These techniques have since been widely adopted for diagnosing and planning the treatment of facial bone reconstruction, particularly in cases involving complex anatomy [16,17]. This results in a virtual 3D model, which allows the surgeon to view the facial structure from different angles and form a mental map to guide the surgical procedures [18]. VSP provides clear visualization of the procedures, helping patients to see the predicted outcomes and ultimately enhancing satisfaction and results [19]. VSP has been well established for mandibular reconstruction workflows, offering notable benefits such as reduced operating room times, lower plate extrusion rates, improved accuracy, and higher rates of bony union [20,21]. However, a recent cost-effectiveness analysis of VSP for mandible reconstruction showed increased costs and higher infection rates compared to conventional free-hand reconstruction [22]. Maxillary reconstruction, being more complex than mandibular reconstruction, may benefit more from VSP. Despite this, VSP for maxillary reconstruction involves a more complicated workflow, greater variability in outcome assessment, and has been less frequently reported. A recent systematic review on the accuracy of VSP for maxillary reconstruction found that incorporating a VSP workflow produced accurate results [23]. This literature review aimed to highlight all available treatment planning techniques for facial bone reconstruction and evaluate the accuracy of VSP compared to traditional methods

## 2. Materials and Methods

### 2.1. Search Strategy

To conduct this review, literature searches were performed using free text and MeSH terms across multiple databases, including Medline, Web of Science, and Scopus, with the help of search engines such as PubMed and Google Scholar. The focus was on studies detailing the planning mechanisms for facial reconstruction operations, with the final search completed on 8 December 2024. On Google Scholar, the search was confined to the top 100 most relevant articles from the past decade. Research was performed to identify optimal search terms. The cited articles covered topics related to facial reconstruction and treatment planning. The keywords and search terms used are presented in Table 1, whereas the details of the search strategy are presented in Table 2.

### 2.2. Inclusion and Exclusion Criteria

The inclusion criteria for this review are randomized clinical trials (RCTs), systematic reviews and meta-analyses, cohort studies, case-control studies, cross-sectional studies, articles published since 1995, and articles published in English. Exclusion criteria were abstracts, author discussions, case reports, editorials (non-peer-reviewed content or opinion pieces), and papers not related to the practical applications of facial bone reconstruction. Studies outside the scope of practical applications in facial bone reconstruction were also excluded, and experimental studies with a patient sample of less than five patients were not included. The included articles provided narrative descriptions of virtual planning in various forms, comparing these methods with conventional methods without specifying a specific study sample size or population. Articles that included interventions on any type of disease or injury were included, whether they were patients with maxillofacial fractures, traumatic injuries, congenital syndromes, or defects resulting from cancer. The primary outcomes focused on enhanced accuracy, improved patient outcomes, and reduced surgical time. Secondary outcomes included cost-effectiveness. There were no limitations on the method used, whether it was VSP, involving the integration of virtual tools for accurate planning of surgical procedures; computer-assisted design (CAD), involving the utilization of CAD for designing surgical guides and implants; computer-aided manufacturing (CAM), involving the implementation of CAM to fabricate custom implants and surgical tools; or stereolithography, a form of 3D printing used to create precise models and guides.

### 2.3. Data Extraction

Titles and abstracts were independently reviewed by the author (M.M.S.) based on the inclusion criteria. The full texts of the selected articles were then analyzed by the authors (M.M.S. and M.N.K.) to determine their suitability for inclusion. Relevant information about virtual or traditional surgical planning (TSP) was extracted and verified by the authors (M.N.K., M.Y.H. and K.M.A.D.).

## 3. Results

**Figure 1:**
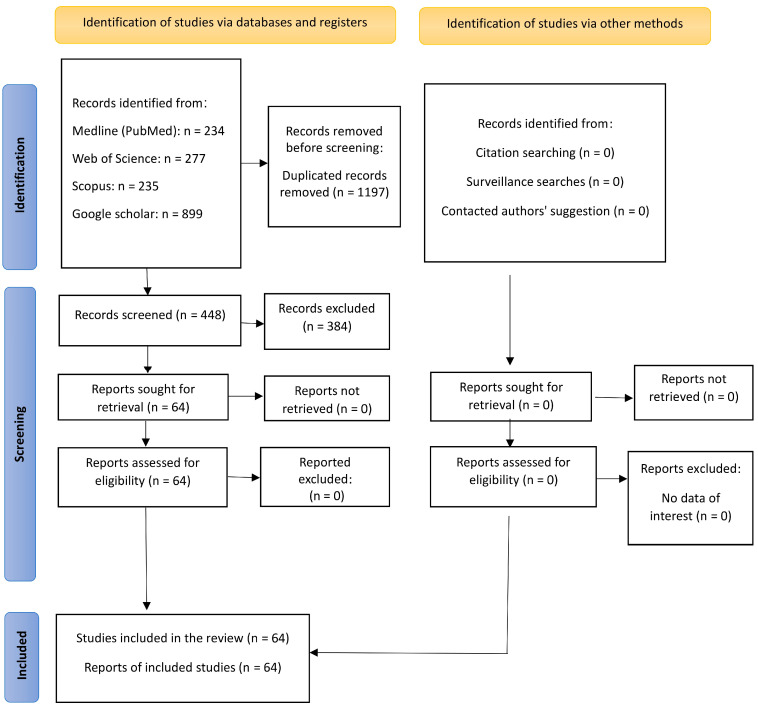
Flow diagram of the reviewing process from study identification and screening to the inclusion of eligible papers in the final report.

There were 1645 potential articles identified. After the removal of 1197 duplicates, 448 titles and abstracts were assessed. Then, 384 papers were excluded because they did not meet the inclusion criteria and were not related to the topic of this review. All the remaining 64 papers were retrieved and analyzed to conduct this review (Figure 1). This topic will be covered through three themes. The first theme includes a presentation of the development of planning methods, transitioning from two-dimensional (2D) methods to 3D virtual methods. The second theme includes an explanation of the progress made in virtual planning. The third theme presents special cases and examples of the use of VSP.

### 3.1. First Theme: Evolution from 2D Freehand Surgical Planning to 3D Virtual Surgical Planning

TSP for facial bone reconstruction involves a series of steps that heavily rely on the surgeon’s experience and 2D imaging techniques [24]. The process begins with a thorough clinical examination of the patient’s facial structure to assess the extent of the injury or defect. Standard radiographs and sometimes CT scans are then used to obtain 2D images of the maxillofacial and mandibular bones, which help the surgeon understand the anatomical loss and plan the reconstruction. Using these images, the surgeon manually plans the surgery, often utilizing physical models or sketches to visualize the reconstruction sites. During the surgery, the surgeon makes real-time adjustments based on visual and tactile feedback from the patient’s anatomy. The next step involves harvesting bone grafts from the patient’s own body (autografts) or using donor bone (allografts) to reconstruct the maxillofacial and mandibular bones. The harvested bone is then shaped and fixed in place using plates, screws, or wires, with the surgeon relying on their expertise to ensure proper alignment and stability [25]. In addition to addressing bone defects, the surgeon also manages any soft tissue defects by using local flaps or free tissue transfers to cover exposed bone and restore facial and mandibular contours. The soft tissues are meticulously sutured to achieve the best possible aesthetic and functional outcome. Postoperative care includes close monitoring for any complications, such as infection, graft failure, or improper healing. The patient may also undergo a rehabilitation program to restore function and aesthetics, which may include physical therapy and follow-up surgeries [25]. However, the traditional techniques have several disadvantages, including limited resolution of the manipulated images and difficulties in interpreting the findings or predicting the outcomes [26]. It is important to note that the success of the surgery heavily depends on the surgeon’s experience and judgment, which can vary [24]. Though still in use, these methods often result in less accurate outcomes [27]. The limitations become evident when dealing with complex anatomical structures and detailed surgical tasks, where precision is crucial [27].

Facial reconstruction has since seen significant advancements, particularly in planning and predicting treatment outcomes. The integration of 3D models and virtual planning has transformed the field, allowing for the creation of precise, patient-specific models using high-resolution imaging techniques like CT scans [8].

Three-dimensional digital planning takes this further by incorporating computer-aided design and manufacturing computer-aided design/computer-aided manufacturing (CAD/CAM) technologies. This integration allows for the design and fabrication of customized surgical guides and implants, tailored to each patient’s unique anatomy [10]. Such precision not only enhances the surgical process but also ensures that implants fit perfectly, leading to better functional and aesthetic results [28]. Effective surgical planning requires precise visualization of the anatomical structure needing reconstruction. Imaging techniques like computed tomography (CT) and magnetic resonance imaging (MRI) are essential diagnostic tools for maxillofacial and mandibular injuries [29]. These techniques complement each other: CT is specialized for hard tissue, while MRI is more useful for soft tissue structures. CT images can be converted into 3D views using software, aiding pre-surgical applications such as planning cuts and creating cutting guides [30,31]. VSP, utilizing CT images and 3D-printed models, has revolutionized reconstructive surgery. This approach allows surgical teams to perform virtual surgeries on 3D models, which is invaluable for educating both patients and surgical teams [32].

#### 3.1.1. Quality and Precision of Digital Planning in Facial Reconstruction

Digital planning in facial reconstruction has revolutionized the field by providing high-precision and personalized treatment plans [33]. Technologies like VSP and 3D printing have significantly improved surgical outcomes [24]. VSP allows for detailed 3D visualization of the surgical site, enabling real-time adjustments and improving preoperative planning [24]. This leads to more accurate and predictable surgical outcomes [24].

#### 3.1.2. CT vs. CBCT Accuracy

CT scans are generally more accurate than cone beam computed tomography (CBCT) for facial reconstruction. CT scans offer higher resolution and better image quality, which are crucial for detailed anatomical reconstructions [34]. CBCT, while useful and less expensive, has lower resolution and may not capture fine details as effectively as CT scans [34].

#### 3.1.3. Printing Technology and Quality

Three-dimensional printing technology plays a significant role in enhancing the quality of facial reconstruction. It allows for the creation of patient-specific surgical guides and implants, which improve surgical precision and reduce operation times [35]. Also, 3D-printed models help surgeons plan and simulate surgeries more accurately, leading to better outcomes [35].

### 3.2. Second Theme: Advances in Facial Reconstructive Surgery Using VSP

Facial region fractures are usually caused by trauma or accidents and can be classified into zygomaticomaxillary complex fractures, Le Fort fractures, and mandibular fractures. The main causes of zygomaticomaxillary complex fractures include sports injuries and car, motor, or fall accidents [36]. These fractures are divided into three main types: type A1 (zygomatic arch fracture), type A2 (lateral orbital wall fracture), type B (fractures involving all four bone sites), and type C (complex fractures extending to the zygomatic bone) [37]. Le Fort fractures commonly occur with midface fractures. They are called hemi-Le Fort fractures when involving one side of the face. Le Fort fractures are divided into three types: Le Fort I (horizontal plane fractures traversing the alveolus of the maxilla from the pyriform sinus), Le Fort II (pyramidal fractures spanning from the maxilla to the orbit), and Le Fort III (complete craniofacial disjunction) [38]. Mandibular fractures, the most common after nasal bone fractures, are classified based on the anatomical structure involved into coronoid process, ramus, condyle, angle, alveolus, body, symphysis, and parasymphysis fractures [39]. Reconstructive surgery in the facial region is challenging due to factors like overlapping soft tissues, difficulty accessing deep bone structures, and potential issues with post-surgical fixators [40]. Inaccuracies during surgery can lead to problems such as facial asymmetry, diplopia, and malocclusion. Incorporating 3D techniques into reconstructive surgery improves pre-surgical evaluation and planning and enables the fabrication of patient-specific appliances and plates to fix bone fragments, enhancing aesthetic outcomes and reducing procedure times [41,42]. Many studies have evaluated the aesthetic and functional results of reconstructive surgery with VSP through clinical evaluations and patient satisfaction surveys [43,44]. By providing detailed, patient-specific data, VSP facilitates better preoperative planning, intraoperative precision, and postoperative results [45]. This leads to reduced surgical times, minimized complications, and enhanced patient satisfaction [45]. Accuracy in VSP refers to the ability of 3D printing and image acquisition techniques to rebuild patient anatomy or design accurate shapes [46]. The 3D models from virtual planning facilitate detailed preoperative simulations, enabling surgeons to visualize and plan each step of the procedure with greater accuracy. VSP techniques are a favorable alternative to in situ plate bending and intraoperative planning [47]. The interest in using technology in reconstructive procedures reduces human errors [48]. Thus, the collected data can be virtually translated into plate bending templates, surgical cutting guides, and reconstruction plates, enhancing the accuracy of applying virtual plans in surgical procedures [49]. Research shows that VSP techniques in facial reconstructive surgery are more accurate, require less operative time, and make surgeons feel more relaxed and confident compared to TSP [50,51,52,53]. For instance, Maglitto et al. reported excellent alignment between postoperative outcomes and preoperative virtual planning in orbital floor fracture surgery using VSP [54]. VSP for jaw relationships improves the alignment of bone grafts, resulting in better dental occlusion and proper jaw relationships [55]. Previously, the success of reconstructive surgery relied on the surgeon’s experience and 2D photographs, which had limitations. Now since most simulation steps are completed before the operation, the total surgical process time is reduced. VSP techniques offer several benefits over TSP, such as better teeth alignment, better bone repositioning, enhanced aesthetic aspects, a tendency to reduce the amount of wasted bone, shorter operation times, and decreased post-surgical complication rates [49,56,57,58]. Reducing operation time with VSP techniques reduces exhaustion and ischemia time [59]. Reduced ischemia time leads to better flap observation and reductions in post-surgical complications [52]. All these advantages of VSP techniques increase intra-surgical efficacy [44].

Prefabricated models also serve as important educational tools for patients and surgical teams [60]. Several studies have found that VSP is more accurate than TSP in postoperative symmetry measurements, including bilateral condylar positions and mandibular angle symmetry [61,62]. However, some studies, like those by Zhang et al. and Bartier et al., found no significant difference between VSP and TSP in certain measurements [63,64]. The initial high cost of VSP steps, such as prefabricated reconstructive plates, might be a concern [65], but the benefits of reduced operation times, complications, and need for surgical re-intervention can make VSP relatively cost-effective [48,66,67].

### 3.3. Third Theme: Examples of Some VSP Clinical Applications

#### 3.3.1. Zygomaticomaxillary Complex Surgeries

Surgeons often enhance operation accuracy by replicating the uninvolved side of the face. Patel et al. concluded that using VSP techniques and prefabricated implants restored both aesthetic and functional aspects in front-orbital and dislocated zygomaticomaxillary complex surgeries [68,69,70].

#### 3.3.2. For Tripod Fractures of the Orbito-Zygomaticomaxillary Complex Management There Were a Few Methods

##### Virtual Reduction Method

This method uses advanced computer technology, such as 3D modeling and VSP, to plan the surgery before it happens. Surgeons use CT scans to create a virtual model of the patient’s skull, allowing them to simulate the reduction and fixation of the fracture. This helps achieve more precise results and reduces the time spent on surgery [69].

##### Mirroring Method

The mirroring method involves creating a mirror image of the unaffected side of the face to guide the reconstruction of the fractured side. By superimposing the healthy side over the fractured side, surgeons can accurately align the bones and ensure symmetry. This method is often used in conjunction with 3D imaging to enhance accuracy [71].

##### Traditional Method

The traditional method, also known as open reduction and internal fixation (ORIF), involves surgically exposing the fracture site to manually realign the bones [72]. Surgeons use plates and screws to stabilize the bones in their correct position. This method relies heavily on the surgeon’s experience and skill to achieve proper alignment and fixation [72]. The virtual reduction method proved the most accurate compared to both the mirroring and traditional methods. Additionally, virtual reduction had the shortest surgical time (89.5 min), followed by mirroring (94.25 min) and traditional approaches (96.75 min) [73].

#### 3.3.3. Mandibular Reconstruction

**Figure 2:**
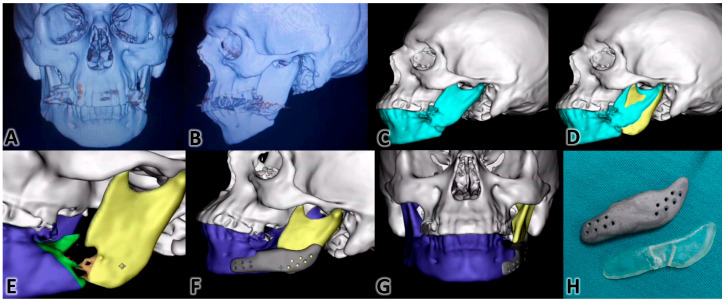
3D VSP. (**A**): CBCT-based 3D rendered model of the patient’s skull in the frontal view showing the misplaced left ramus due to the bone loss at the gonial angle of the mandible. (**B**): The lateral view of the 3D model shows the location of the bone loss. (**C**): The case after being transferred to the 3D simulation software. (**D**): Modification of the distal segment of the mandible in 3D. (**E**): Refining the contours before designing the replacement portion. (**F**): The gonial prosthesis is in place with extensions carrying holes for the fixation screws. (**G**): Frontal view of the prosthesis in place. (**H**): The 3D-printed titanium prosthesis accompanied with a 3D-printed resin surgical guide (Credit: M.M.S. and K.M.A.D., Oral and Maxillofacial Surgery Department).

**Figure 3:**
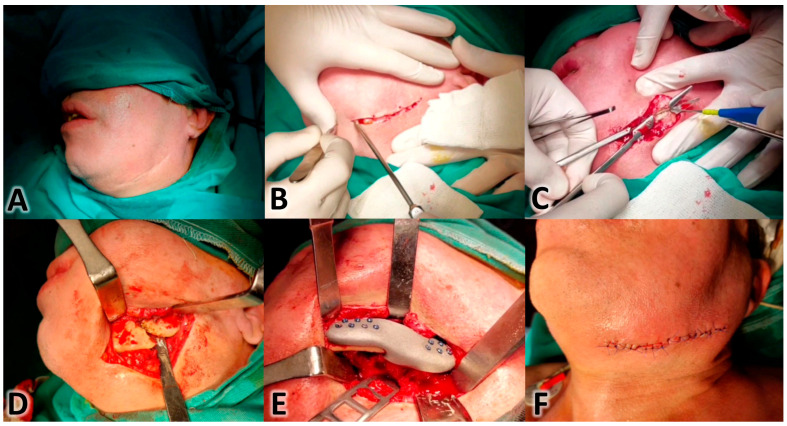
The surgical intervention following the 3D VSP. (**A**): Intra-operative facial photograph of the surgical area where the reconstruction of the mandible will take place. (**B**): Surgical access under the mandibular border to expose the left gonial area. (**C**): Entrance to the surgical site in layers until reaching the bone loss area. (**D**): The location of the bone loss at the gonial angle of the mandible. (**E**): The 3D-printed metallic prosthesis was inserted into its proper place after adjusting the spatial positions of the mesial and distal segments according to the VSP. (**F**): The final appearance of the surgical area following suturing (Credit: M.M.S. and K.M.A.D., Oral and Maxillofacial Surgery Department, Faculty of Dentistry, University of Damascus).

Using VSP in mandibular reconstruction offers several benefits, such as increased operation efficacy and reduced total procedure time, by utilizing premade guides and plates [66]. Studies by Hanasono et al. and Dessoky et al. noted significant decreases in operation times and the accurate fitting of prefabricated plates, respectively [65,74], while Möllmann et al. found no significant time difference between VSP and TSP. However, the overall consensus is that VSP saves time and cost [75]. With the use of pre-formed plates and guides, the need for intraoperative adjustments is greatly reduced. This leads to a more streamlined and quicker surgery [76], Additionally, patient-specific implants negate the necessity of a separate donor site. Three-dimensionally printed titanium cribs or meshes not only offer strength and durability but also include features that facilitate bone graft placement and promote excellent dental rehabilitation [77]. The stages of the virtual planning of the mandibular angle and body reconstruction case are shown in Figure 2. The surgical implementation of this planning is shown in Figure 3. This case was treated by two co-authors of this paper (M.M.S. and K.M.A.D). Patient informed consent for the use of his images in a medical publication was obtained.

#### 3.3.4. Hemifacial Microsomia

Traditional treatment for hemifacial microsomia involves orthognathic surgery and mandibular distraction osteogenesis [78,79,80]. Virtual osteotomy positions for mandibular distraction osteogenesis are customized for each patient. The location and direction of the distractors are adjusted multiple times until the ideal status is achieved [81].

#### 3.3.5. Soft Tissue Correction

After facial bone reconstructive surgery, soft tissue correction is also required. The most common technique used is autologous fat grafting due to its effectiveness, simplicity, and cost-effectiveness. VSP is employed to assess the location and amount of fat to be injected [82].

#### 3.3.6. Fabrication of Intraoperative Guidance

In cases of facial fractures, guide plate design can simulate the fracture region along with the location and shape of the prefabricated titanium plate. This enhances the primary fixation and positioning of the guide plate [83,84]. This intraoperative simulation system aids in achieving accurate bone repositioning and implant placement, thereby decreasing operation time [85,86].

## 4. Discussion

### 4.1. Efficiency VSP in Facial Reconstructive Surgery

The accuracy and benefits of digital planning in facial reconstruction are well-documented. VSP and 3D printing are transforming the field by delivering high-precision and personalized treatment plans. CT scans typically provide higher resolution and superior image quality, which are essential for detailed anatomical reconstructions. In contrast, cone beam computed tomography (CBCT), while useful and cost-effective, generally has lower resolution. This discrepancy can be attributed to the fact that CBCT images are often affected by noise and artifacts, which degrade image quality [87].

The role of 3D printing in enhancing the quality of facial reconstruction is well-documented. Three-dimensionally printed models significantly improve surgical precision and reduce operation times by providing patient-specific surgical guides and implants. Research widely supports the advantages of VSP, highlighting its superiority in reducing surgical time, minimizing complications, and enhancing patient satisfaction. VSP allows for detailed preoperative simulations, enabling surgeons to visualize and plan each step of the procedure with greater accuracy. This, in turn, reduces the time spent in the operating room and facilitates the creation of patient-specific surgical guides and implants through 3D printing.

An important aspect of VSP’s effectiveness is its educational value. VSP provides valuable educational tools for both patients and surgical teams. The ability to visualize the surgical plan and understand the steps involved helps set realistic expectations and improves communication between the surgeon and the patient.

### 4.2. Applicability of VSP in Facial Reconstructive Surgery

In zygomaticomaxillary complex surgeries, surgeons can improve operation accuracy by replicating the uninvolved side of the face. VSP helps in achieving precise reconstruction and enhances patient outcomes [69].

In the management of tripod fractures of the zygomatic and maxillary complex, surgeons can utilize CT scans to create a virtual model of the patient’s skull and simulate the reduction and fixation of the fracture using the virtual reduction method. This approach has proven to achieve more accurate results and reduce the time spent in surgery. The mirroring method, benefiting from 3D imaging, ensures symmetry and accuracy. Conversely, the traditional method, which involves surgically exposing the fracture site to manually realign the bones using plates and screws for fixation, relies heavily on the surgeon’s experience and skill. In conclusion, VSP offers greater intraoperative accuracy, reduced surgical times, and fewer postoperative complications compared to traditional surgery, with virtual reduction being the most effective method [73].

In mandibular reconstruction, the consensus is that the VSP technique saves time and costs by using ready-made plates and guides. This reduces the need for adjustments during surgery and eliminates the need for a separate donor site [76].

In soft tissue correction, the effectiveness of VSP is evident in assessing the location and amount of fat to be injected to correct soft tissue after reconstructive surgery. By leveraging VSP in these applications, surgeons achieve greater precision, reduced surgical times, and improved patient outcomes in various complex facial surgeries [82].

### 4.3. Future Research

An accurate estimate of all the above factors is required, including the time spent and initial costs of VSP; overall healthcare utilization costs, including for complications and their management for both VSP and TSP; follow-up periods and the number of visits; other concomitant surgeries needed to achieve full restoration of function and appearance; and the length of hospital stays and overnight stays, which may be associated with complications and treatment costs. Additionally, there is a lack of evidence in the literature regarding the characteristics that assess the success of VSP programs, which hinders the generalizability of findings.

### 4.4. Limitations

The limitations of this study are the absence of a standardized protocol and the reliance on the authors’ perspectives, which introduces subjectivity and increases the risk of bias due to the interpretation and selection of articles. However, the strengths of this study lie in its ability to provide a broad understanding of the topic by summarizing numerous articles and offering deep insights into the context and background of virtual planning. This flexible approach to literature review, free from a strict protocol, proves valuable in comprehending these complex issues.

## 5. Conclusions

VSP enhances the prediction of aesthetic and functional outcomes in reconstructive surgery, addressing complex injuries from trauma, oncology, and orthognathic procedures. Using sophisticated imaging technologies and CAD/CAM, VSP creates precise plans that reduce human errors, increase surgical precision, and improve patient satisfaction and recovery. Additionally, VSP allows for patient-specific guides and templates, minimizing intraoperative adjustments, reducing operation time, and improving implant integration through 3D printing. This technological advancement significantly improves reconstructive surgery.

## Figures and Tables

**Table 1:** Keywords used in the search.

Treatment plan	facial bone reconstruction, facial fractures, hemifacial microsomia, facial bone grafting, osteotomies, augmentation, replacement, grafting, breakages, and jaw replacement.
Surgical planning tools	virtual surgical planning, computer-assisted methods, free-hand methods, traditional surgical planning, 3D digital planning, 2D methods,
Outcomes	accuracy, satisfaction, operation time, cost, discomfort, stabilization, harms, recurrence, untoward effects.

**Table 2:** Electronic search strategy.

PubMed Publication date: Up to 8 December 2024 Search builder: All fields	#1 (facial bone reconstruction “OR” facial fractures “OR” hemifacial microsomia “OR” facial bone grafting “OR” osteotomies “OR” augmentation “OR” replacement “OR” grafting “OR” breakages “OR” jaw replacement) #2 (virtual surgical planning “OR” computer-assisted methods “OR” free-hand methods “OR” traditional surgical planning “OR” 3D digital planning “OR” 2D methods) #3 (accuracy “OR” satisfaction “OR” operation time “OR” cost “OR” discomfort “OR” stabilization “OR” harms “OR” recurrence “OR” untoward effects) #4 #1 AND #2 AND #3
Web of Science	#1TS= (facial bone reconstruction OR facial fractures OR hemifacial microsomia OR facial bone grafting OR osteotomies OR augmentation OR replacement OR grafting OR breakages OR jaw replacement) #2TS= (virtual surgical planning OR computer-assisted methods OR free-hand methods OR traditional surgical planning OR 3D digital planning OR 2D methods) #3TS= (accuracy OR satisfaction OR operation time OR cost OR discomfort “OR” stabilization OR harms OR recurrence OR untoward effects) #4 #1 AND #2 AND #3
Scopus Publication date: Up to 8 December 2024	#1 TITLE ABS-KEY 1 (facial bone reconstruction “OR” facial fractures “OR” hemifacial microsomia “OR” facial bone grafting “OR” osteotomies “OR” augmentation “OR” replacement “OR” grafting “OR” breakages “OR” jaw replacement) #2 TITLE ABS-KEY (virtual surgical planning “OR” computer-assisted methods “OR” free-hand methods “OR” traditional surgical planning “OR” 3D digital planning “OR” 2D methods) #3 TITLE ABS-KEY (accuracy “OR” satisfaction “OR” operation time “OR” cost “OR” discomfort “OR” stabilization “OR” harms “OR” recurrence “OR” untoward effects) #4 #1 AND #2 AND #3
Google Scholar	(facial bone reconstruction “OR” facial fractures “OR” hemifacial microsomia “OR” facial bone grafting “OR” osteotomies “OR” augmentation “OR” replacement “OR” grafting “OR” breakages “OR” jaw replacement) AND (virtual surgical planning “OR” computer-assisted methods “OR” free-hand methods “OR” traditional surgical planning “OR” 3D digital planning “OR” 2D methods) AND (accuracy “OR” satisfaction “OR” operation time “OR” cost “OR” discomfort “OR” stabilization “OR” harms “OR” recurrence “OR” untoward effects)

## Data Availability

The data presented in this study are available upon request from the corresponding author.

## Abbreviations

The following abbreviations are used in this manuscript:

VSP	virtual surgical planning
TSP	traditional surgical planning
2D	two-dimensional
3D	three-dimensional
CT	computed tomography
